# Case Report: Management of a congenital intrahepatic portosystemic shunt with portal vein aneurysm in a child using 3D computer-assisted partial right hepatectomy

**DOI:** 10.3389/fped.2024.1429537

**Published:** 2024-10-28

**Authors:** Yao Liu, Johan Arief, Wenli Xiu, Xiwei Hao, Feifei Wang, Nan Xia, Qian Dong

**Affiliations:** ^1^ Department of Pediatric Surgery, The Affiliated Hospital of Qingdao University, Qingdao, China; ^2^ Shandong Provincial Key Laboratory of Digital Medicine and Computer-Assisted Surgery, Qingdao, China; ^3^ Institute for Digital Medicine and Computer-Assisted Surgery in Qingdao University, Qingdao, China

**Keywords:** Congenital Intrahepatic Portosystemic Shunts, aneurysm, 3D computer assisted surgery, case report, partial right hepatectomy

## Abstract

Congenital portosystemic shunts (CPSS) are rare pediatric vascular malformations characterized by abnormal development of the portal vein, which is attributed to incomplete embryonic remodeling of the hepatic and surrounding vasculature. CPSS manifests in two main forms: intrahepatic and extrahepatic. This study details the management of a pediatric patient diagnosed with Congenital Intrahepatic Portosystemic Shunt (CIPS) who was referred to our institution. By using a computer-assisted surgical system, the right hepatectomy was successfully performed, guided by precise intraoperative navigation based on three-dimensional reconstructions of enhanced CT imagery. The patient exhibited a favorable postoperative recovery trajectory, with the absence of complications or recurrence throughout the monitoring period.

## 1 Introduction

Congenital portosystemic shunts (CPSS) are rare vascular anomalies that create an abnormal connection between the portal and systemic circulations. The incidence of CPSS is estimated to be 1 in 30,000–50,000 live births (1, 2). A subset of CPSS, Congenital Intrahepatic Portosystemic Shunts (CIPS), specifically occurs within hepatic tissue (3). CIPS enables the diversion of a segment of portal venous blood directly into the systemic venous system, which can lead to complications such as hypergalactosemia and hyperammonemia. In severe cases, these complications can progress into hepatic encephalopathy (4). The rare occurrence of CIPS contributes to ongoing debates and uncertainties within the medical community regarding the most optimal therapeutic strategies (3).

Abdominal contrast-enhanced computed tomography (CT) imaging is a crucial diagnostic tool for CIPS, with enhanced CT scans offering substantial efficacy in identifying shunts and detecting coexisting hepatic and extrahepatic abnormalities (5). The Hisense Computer-Assisted Surgery (CAS) system enhances this diagnostic ability by transforming two-dimensional CT images into detailed three-dimensional reconstructions. This advanced technology precisely outlines the anatomical positioning and variations of intrahepatic vasculature, alongside providing a comprehensive spatial representation of lesions relative to surrounding tissues and organs (6). The application of CAS in three-dimensional imaging plays a pivotal role in formulating personalized treatment strategies for pediatric patients, thereby potentially improve their overall prognosis.

This case report outlines the clinical journey of a child with CIPS treated at our institution, highlighting the implementation of the Hisense CAS system for preoperative three-dimensional CT reconstruction.

## 2 Case report

### 2.1 Patient history

A 10-year-old male patient presented with abdominal pain at a local healthcare facility. Ultrasonographic evaluation at the initial medical center revealed the presence of a portal-hepatic venous fistula. Subsequently, a referral to a tertiary care hospital was recommended for advanced management. Upon presentation at our institution, the patient underwent a series of comprehensive diagnostic assessments.

### 2.2 Laboratory evaluations

Serological examination revealed elevated levels of alkaline phosphatase (170 U/L; reference range: 45–125 U/L), glycocholic acid（56.39 μg/ml; reference range: 0–2.7 μg/ml, and total bile acids (99.03 μmol/L; reference range: 0–12.0 μmol/L). Additionally, the blood ammonia level was elevated at 64 µmol/L (reference range: 10.0–47.0 µmol/L).

However, the total bilirubin, direct bilirubin, indirect bilirubin, alanine aminotransferase (ALT), aspartate aminotransferase, and glutamyl transpeptidase were all within normal reference values.

### 2.3 Radiological assessment

**Figure 1:**
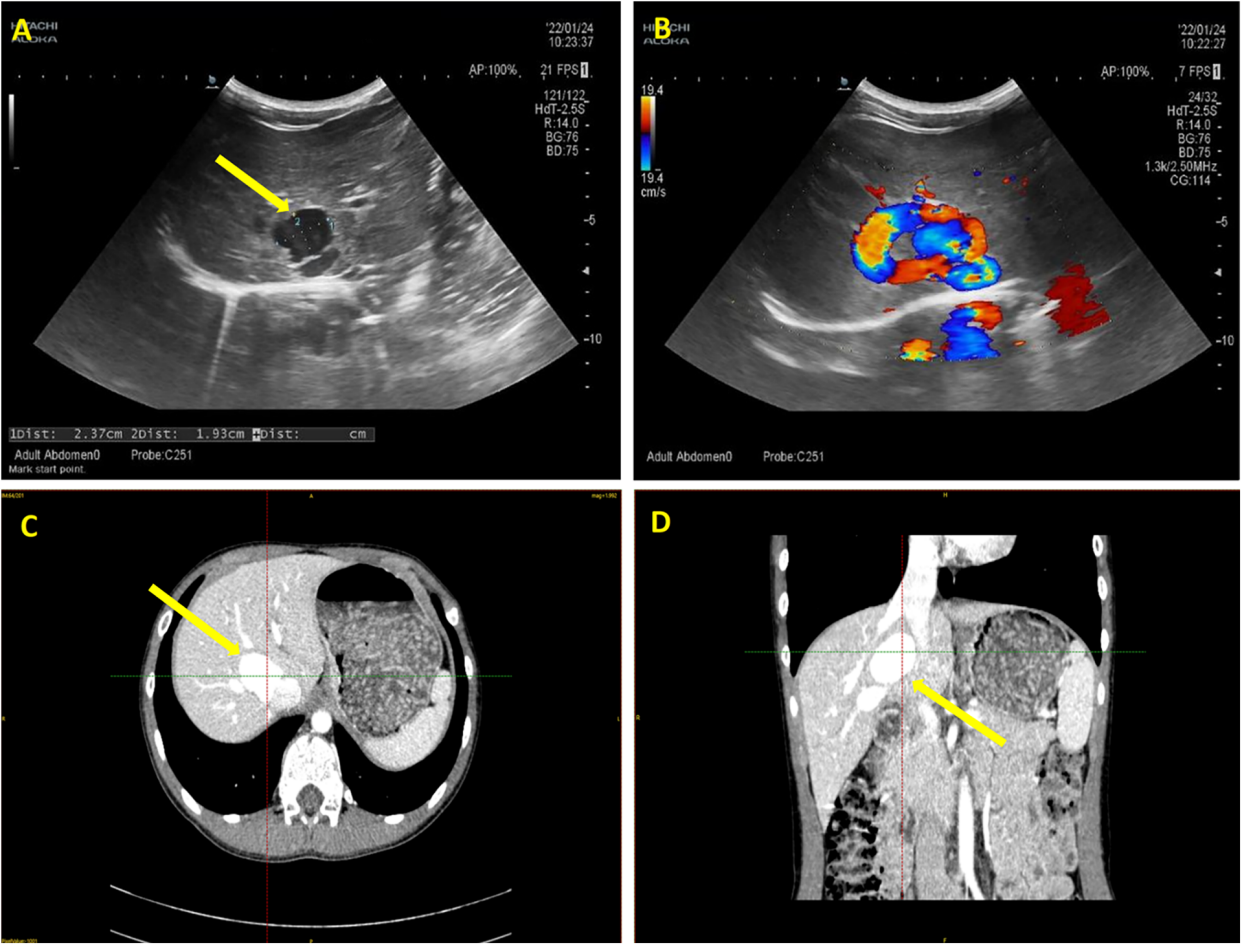
Ultrasonography and enhanced computed tomography (CT) imaging of the patient abdomen. **(A)** The arrow highlights the tumor-like dilatation of the portal vein, demarcated by distinct boundaries. **(B)** Color Doppler ultrasound imaging verifies the directional flow from the portal vein to the inferior vena cava, with the flow velocity of 68.0 cm/s. **(C)** Transverse section and **(D)** Coronal section of enhanced CT images. The arrows indicate the tortuously dilated portal vein.

The patient underwent diagnostic abdominal ultrasonography at our facility. Imaging findings revealed portal venous inflow into the liver (Figure 1). The portal vein's main trunk and the proximal segment of its right branch both measured approximately 0.8 cm in diameter. Notably, a tortuous course was observed posteriorly and medially towards the inferior vena cava, culminating in a tumor-like dilatation measuring 2.4 × 1.9 cm (Figure 1A) at the vicinity of the inferior vena cava, establishing a communication channel approximately 0.6 cm wide. Color Doppler Imaging (CDPI) demonstrated vortex flow within this portal vein expansion (Figure 1B). Pulsed Wave (PW) Doppler analysis revealed a tortuous course of the proximal segment of the right branch of the portal vein, deviating posteriorly and medially, with aneurysmal dilation near the inferior vena cava and communication with it. The blood flow spectrum in the channel showed characteristics intermediate between the portal vein and the inferior vena cava, with a flow velocity of 68.0 cm/s. No other abnormalities were detected in the abdominal viscera.

To gain a more comprehensive understanding of the anatomical positioning and trajectory of the tumor-like dilated portal vein, an enhanced computed tomography (CT) scan with subsequent three-dimensional reconstruction was advised. The dynamic contrast-enhanced CT of the upper abdomen revealed a convoluted enlargement of the right branch of the portal vein, measuring approximately 2.5 cm in diameter. Notably, this segment exhibited a local communication with the intrahepatic portion of the inferior vena cava (Figures 1C,D).

**Figure 2:**
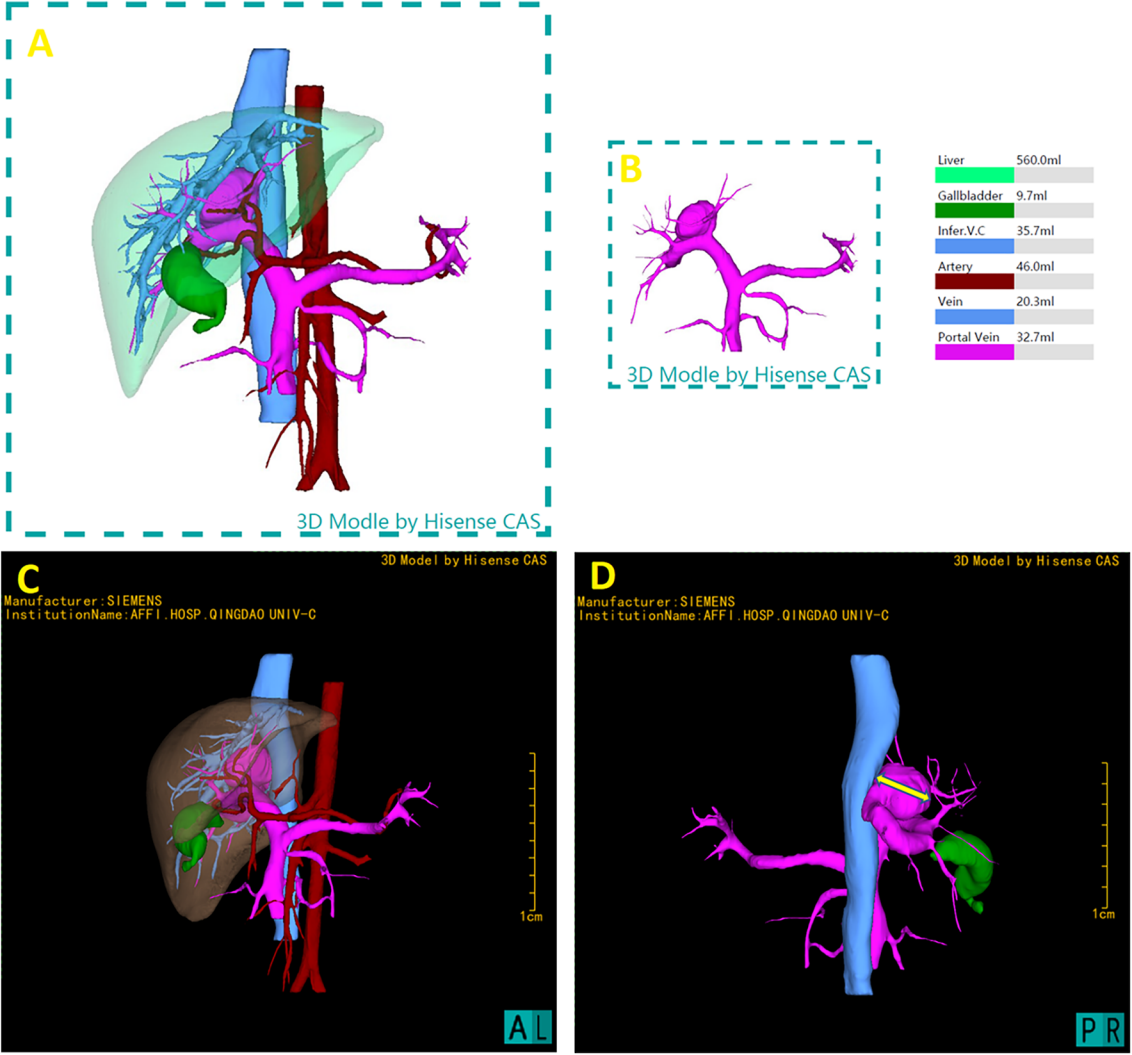
3D reconstruction utilizing the hisense computer-assisted surgery (CAS) system and preoperative three-dimensional model. **(A)** Full reconstruction of liver and surrounding vasculature, showing liver in transparent, measured 560.0 ml **(B)** portal vein measured 32.7 ml, including the aneurysm where the shunt located. **(C)** Represent the lateral perspectives of the 3D reconstruction, showcasing the liver in a semi-transparent state to clarify its internal anatomical structures and vascular variances. **(D)** The measurement of the maximum transverse diameter of the portal vein's tumor-like dilatation, measured as 26.36 mm, providing critical spatial dimensions.

The patient underwent abdominal CT enhancement scans. The DICOM-formatted enhanced computed tomography (CT) images of the patients were integrated into the Hisense Computer-Assisted Surgery (CAS) system for segmentation, pattern identification, denoising, smoothing, and 3D reconstruction. Facilitating the creation of interactive, stereoscopic three-dimensional representations. Hisense CAS system successfully reconstructed the 3D model of liver and were used as navigator tools in the operation room during portosystemic shunt surgery (Figure 2).

This three-dimensional reconstruction provided visual assessment of the shunt's dimensions and anatomical positioning, as well as its spatial relationship with adjacent vascular structures and critical surgical landmarks. 3D Hisense CAS reconstruction facilitates the exploration of the complex liver morphology, enabling the individualized and optimal surgical strategies for this pediatric patient. Using the CAS with the liver hidden, the transverse diameter of aneurysm at the location of shunt was 26.36 mm.

### 2.4 Therapeutic intervention

Considering the patient's elevated blood ammonia levels, the presence of a 1.5 cm wide shunt between the right posterior branch of the portal vein (P7) and the short hepatic vein with a flow velocity of 68.0 cm/s, and vortex flow within the dilated portal vein, interventional therapy was assessed as inadequate and associated with higher risk of thrombosis. Consequently, a surgical ligation plan was initially chosen.

Intraoperatively, a substantial venous aneurysm was identified between the right posterior branch of the portal vein (P7) and the short hepatic vein, characterized by vague boundaries and an indistinct gap. During mobilization, the aneurysm abruptly ruptured, causing a considerable amount of dark red hemorrhage. The aneurysm wall was repaired, and 2 units of red blood cells were transfused. Due to the aneurysm's substantial size, fragile and thin walls, and ineffective hemostasis from suturing, complete excision and ligation were unfeasible. Following consultation with the patient's family, a decision was made to perform partial right hepatectomy and aneurysm resection.

**Figure 3:**
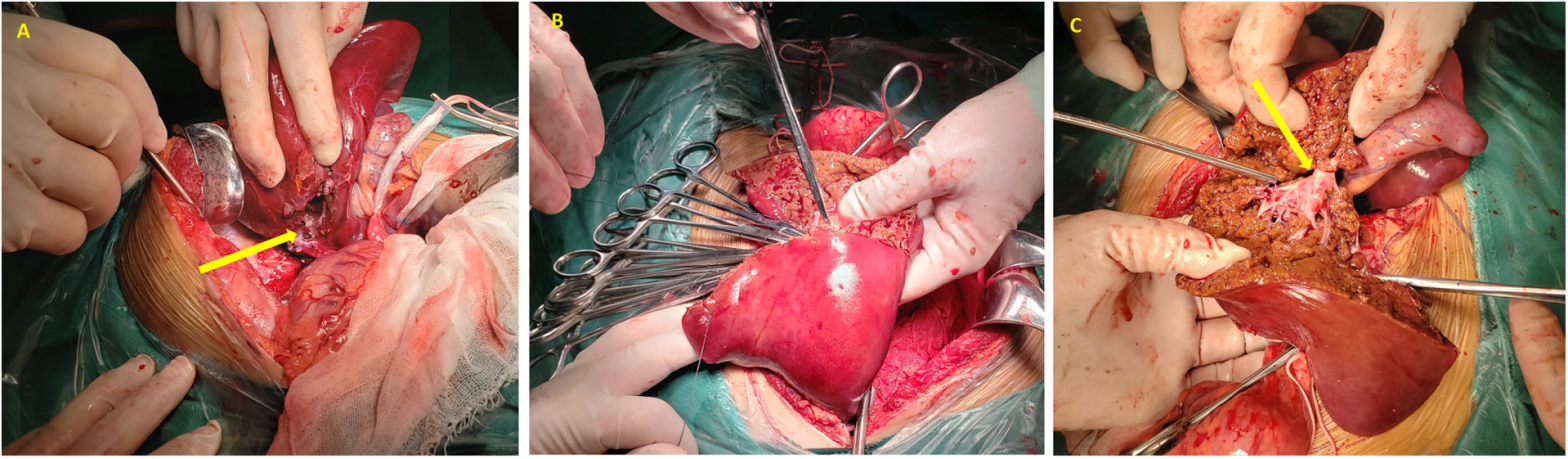
Intraoperative photographic documentation. **(A)** The arrow highlights the ruptured aneurysm; **(B)** hemostasis achieved at the hepatic transection site; **(C)** the arrow identifies the right hepatic vein.

The coronary ligament was mobilized, and the right hepatic vein was ligated. The right liver was mobilized, and the structures at the hepatic transection were sequentially clamped, divided, and ligated, removing the aneurysm and segments V, VI, and VII of the right liver. Hemostasis was achieved at the transection site by suturing and hemostatic dressings, followed by closure of the surgical wound. The abdominal cavity was irrigated with warm saline to ensure hemostasis, and the coronary, right triangular, and falciform ligaments were sutured. A drainage tube was placed adjacent to the hepatic transection and exteriorized through a right abdominal incision (Figure 3).

The patient's total intraoperative fluid infusion was 2,860 ml, including 2,500 ml of crystalloid solution. The estimated blood loss was approximately 300 ml, with a transfusion of 2 units of red blood cells. The postoperative blood pressure was recorded at 122/67 mmHg.

### 2.5 Postoperative recovery

**Figure 4:**
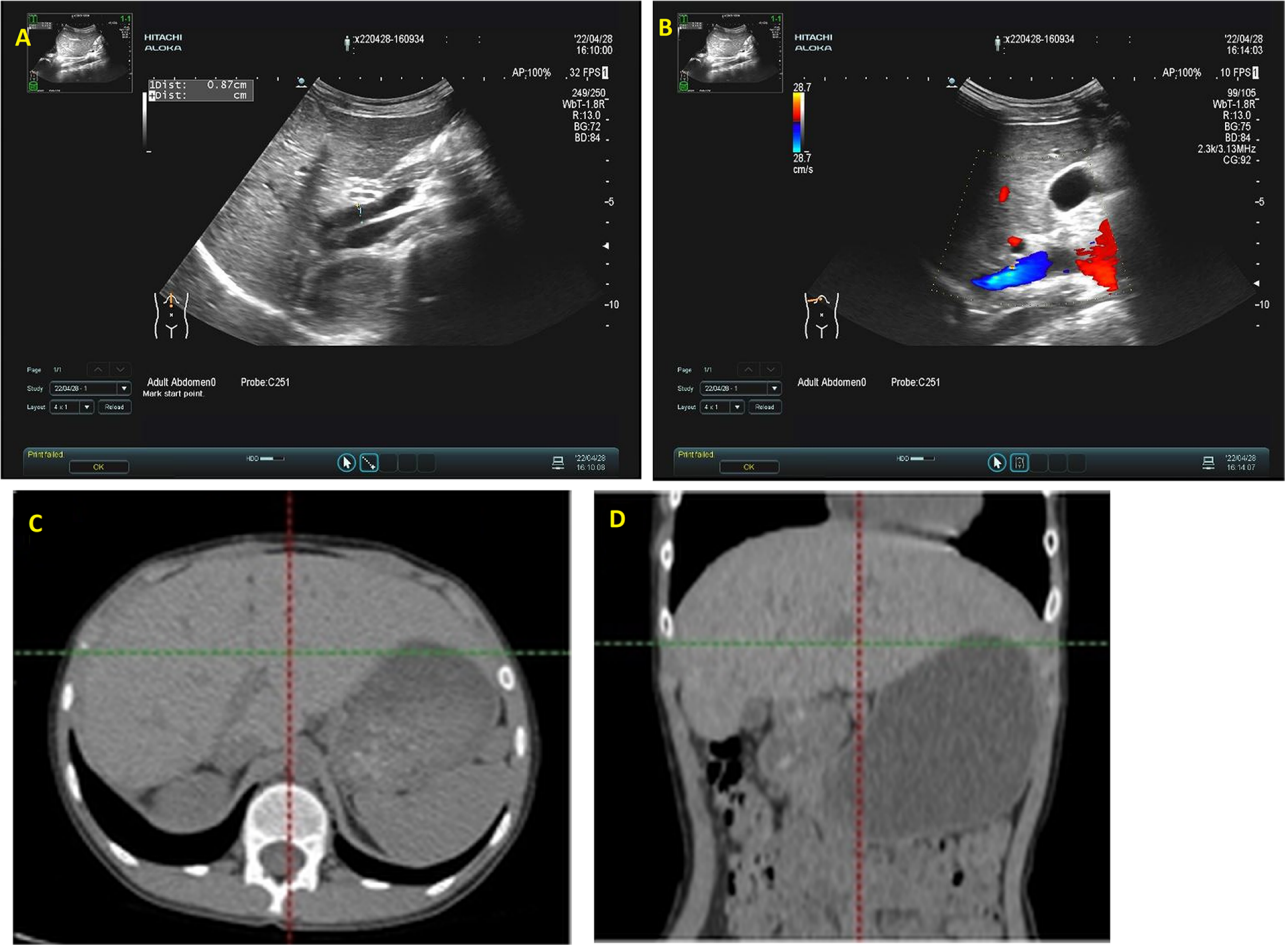
Abdominal ultrasound evaluation and CT examination two months postoperatively. **(A)** Demonstrates robust blood flow within the intrahepatic portal vein, with no notable abnormal echogenicity; **(B)** reveals no identifiable communication between the portal vein and the inferior vena cava. **(C,D)** The liver has regenerated, multiple small nodular shadows can be seen in the lower abdominal cavity.

Postoperative ultrasonography revealed the main trunk of portal vein measuring 0.9 cm in diameter with a flow velocity of 36.6 cm/s, and the inferior vena cava measuring 0.8 cm in diameter with a flow velocity of 42.6 cm/s, respectively. Two months after surgery, the structure of the surgical area is clearer than before (Figure 4). The patient exhibited favorable recovery post-surgery, with subsequent evaluations indicating that both blood ammonia levels and liver function parameters were within normal ranges.

## 3 Discussion

In this case, we conducted a hepatic resection on a pediatric patient with CIPS complicated by hyperammonemia. CPSS represents a rare vascular anomaly, precipitating a spectrum of complications such as hyperammonemia, increased bile acids, hepatic encephalopathy, and hepatic neoplasms. The Park classification system delineates CIPS as follows: Type I involves a singular, substantial communicative branch connecting the right branch of the portal vein to the intrahepatic inferior vena cava; Type II is characterized by single or multiple communications between portal and hepatic vein branches within a specific liver segment; Type III is an extension of Type II with the presence of a venous aneurysm; Type IV encompasses multiple portal vein-hepatic vein communications across several liver segments; Type V involves a persistent venous duct linking the initial segment of the left portal vein branch to the inferior vena cava (7). The patient in this report was classified as Type III.

The initial documentation of CIPS was made by Doehner et al. in 1956 (8). Subsequent advancements in prenatal imaging techniques and enhanced recognition of CIPS have led to an increase in reported cases (9). Ultrasonography is typically employed as the primary imaging modality for CIPS diagnosis (2, 10, 11). However, its efficacy is limited by factors such as restricted imaging characteristics and interference from gastrointestinal gases, hindering accurate delineation of associated intrahepatic and/or extrahepatic shunts. Consequently, in the case presented herein, the patient underwent an upper abdominal CT with dynamic contrast enhancement for a more comprehensive assessment. Contrast-enhanced CT not only facilitates the detection of shunts and delineates the anatomy and positioning of other abdominal vasculature but also aids in identifying lesions and additional anomalies in abdominal solid organs (10). In this case, the patient's contrast-enhanced CT images were integrated into the Hisense CAS system, enabling the creation of interactive, three-dimensional visualizations. This process accurately reconstructed the complex three-dimensional anatomy of the dilated, tortuous portal vein and its adjacent vascular structures. By rendering the liver in a semi-transparent or fully transparent state, the system elucidated the intricate internal anatomical relationships and spatial variations within the hepatic vasculature. This advanced visualization technique facilitated precise calculations of vascular territories for supply or drainage, liver volumetrics, and other critical data unattainable through conventional two-dimensional CT imaging. Such detailed insights are instrumental in aiding clinicians to develop comprehensive clinical diagnostic and therapeutic strategies.

Hyperammonemia and increased bile acid levels are frequently observed laboratory anomalies in CPSS patients. Plasma ammonia concentrations in these patients typically range from 1.1 to 10 times above the standard reference values and are frequently correlated with neurological manifestations (12–14). The pediatric patient discussed in this report exhibited elevated levels of both blood ammonia and bile acids. Prolonged exposure to elevated ammonia levels poses a significant risk of neurotoxicity, particularly detrimental to the developing brain.

The rarity of CIPS and a lack of case studies have limited the establishment of a standardized treatment regimen (15). Therapeutic approaches are dictated by factors such as the shunt's type, anatomical location, functional impact, patient age, and the severity of associated symptoms and complications (3). In younger, asymptomatic patients, CIPS may resolve spontaneously, warranting a conservative management approach (16). Current literature advocates for early intervention in children over 2 years of age or those manifesting clinical symptoms. Interventional therapies, surgical procedures, and liver transplantation are recognized as effective modalities for CIPS management (2, 17, 18). In the presented case, the patient was of an older age group and exhibited no indications of spontaneous shunt closure. Three-dimensional imaging delineated a shunt between the right branch of the portal vein and the inferior vena cava, with the portal vein's tumor-like dilation measuring approximately 26.36 mm at its widest point. The presence of vortex flow within this dilated segment indicated a heightened risk of thrombus formation. After thorough consideration of the patient's unique clinical scenario and obtaining consent from the family, a surgical intervention was deemed the most appropriate course of action. The initial surgical plan involved occluding the shunt; however, intraoperative findings revealed the substantial size and fragile nature of the vessel walls at the site of the portal vein's aneurysmal change, precluding complete resection.

CIPS are rare but can lead to severe complications. Ultrasound is the primary diagnostic tool, supplemented by contrast-enhanced CT for detailed shunt analysis. Three-dimensional imaging aids in visualizing hepatic vascular structures, supporting targeted treatment planning. In cases with significant portal vein alterations, partial hepatectomy may be necessary, helping to reduce recurrence risks and improve patient outcomes.

## Data Availability

The original contributions presented in the study are included in the article/Supplementary Material, further inquiries can be directed to the corresponding authors.
